# Single Point Insulin Sensitivity Estimator in Pediatric Non-Alcoholic Fatty Liver Disease

**DOI:** 10.3389/fendo.2022.830012

**Published:** 2022-02-02

**Authors:** Dieter Furthner, Christian-Heinz Anderwald, Peter Bergsten, Anders Forslund, Joel Kullberg, Håkan Ahlström, Hannes Manell, Iris Ciba, Harald Mangge, Katharina Maruszczak, Pia Koren, Sebastian Schütz, Susanne Maria Brunner, Anna Maria Schneider, Daniel Weghuber, Katharina Mörwald

**Affiliations:** ^1^Department of Pediatrics, Salzkammergutklinikum Voecklabruck, Voecklabruck, Austria; ^2^Obesity Research Unit, University Hospital Salzburg, Paracelsus Medical University, Salzburg, Austria; ^3^Division of Endocrinology and Metabolism, Department of Internal Medicine III, Medical University of Vienna, Vienna, Austria; ^4^Direction, Arnoldstein Healthcare Centre, Arnoldstein, Austria; ^5^Department of Medical Cell Biology, Uppsala University, Uppsala, Sweden; ^6^Department of Women’s and Children’s Health, Uppsala University, Uppsala, Sweden; ^7^Department of Surgical Sciences, Radiology, Uppsala University, Uppsala, Sweden; ^8^Clinical Institute of Medical and Chemical Laboratory Diagnosis, Medical University of Graz, Graz, Austria; ^9^Department of Pediatrics, University Hospital Salzburg, Paracelsus Medical University, Salzburg, Austria; ^10^Research Program for Receptor Biochemistry and Tumor Metabolism, Department of Pediatrics, University Hospital of the Paracelsus Medical University, Salzburg, Austria

**Keywords:** insulin resistance, pediatric obesity, hepatic insulin resistance index, HOMA-IR, receiver-operating-characteristic curve

## Abstract

**Background:**

Attenuated insulin-sensitivity (IS) is a central feature of pediatric non-alcoholic fatty liver disease (NAFLD). We recently developed a new index, single point insulin sensitivity estimator (SPISE), based on triglycerides, high-density-lipoprotein and body-mass-index (BMI), and validated by euglycemic-hyperinsulinemic clamp-test (EHCT) in adolescents. This study aims to assess the performance of SPISE as an estimation of hepatic insulin (in-)sensitivity. Our results introduce SPISE as a novel and inexpensive index of hepatic insulin resistance, superior to established indices in children and adolescents with obesity.

**Materials and Methods:**

Ninety-nine pubertal subjects with obesity (13.5 ± 2.0 years, 59.6% males, overall mean BMI-SDS + 2.8 ± 0.6) were stratified by MRI (magnetic resonance imaging) into a NAFLD (>5% liver-fat-content; male n=41, female n=16) and non-NAFLD (≤5%; male n=18, female n=24) group. Obesity was defined according to WHO criteria (> 2 BMI-SDS). EHCT were used to determine IS in a subgroup (n=17). Receiver-operating-characteristic (ROC)-curve was performed for diagnostic ability of SPISE, HOMA-IR (homeostatic model assessment for insulin resistance), and HIRI (hepatic insulin resistance index), assuming null hypothesis of no difference in area-under-the-curve (AUC) at 0.5.

**Results:**

SPISE was lower in NAFLD (male: 4.8 ± 1.2, female: 4.5 ± 1.1) than in non-NAFLD group (male 6.0 ± 1.6, female 5.6 ± 1.5; P< 0.05 {95% confidence interval [CI]: male NAFLD 4.5, 5.2; male non-NAFLD 5.2, 6.8; female NAFLD 4.0, 5.1, female non-NAFLD 5.0, 6.2}). In males, ROC-AUC was 0.71 for SPISE (P=0.006, 95% CI: 0.54, 0.87), 0.68 for HOMA-IR (P=0.038, 95% CI: 0.48, 0.88), and 0.50 for HIRI (P=0.543, 95% CI: 0.27, 0.74). In females, ROC-AUC was 0.74 for SPISE (P=0.006), 0.59 for HOMA-IR (P=0.214), and 0.68 for HIRI (P=0.072). The optimal cutoff-level for SPISE between NAFLD and non-NAFLD patients was 5.18 overall (Youden-index: 0.35; sensitivity 0.68%, specificity 0.67%).

**Conclusion:**

SPISE is significantly lower in juvenile patients with obesity-associated NAFLD. Our results suggest that SPISE indicates hepatic IR in pediatric NAFLD patients with sensitivity and specificity superior to established indices of hepatic IR.

## Introduction

The prevalence of non-alcoholic fatty liver disease (NAFLD) in children and adolescents is on the rise, hence emerging as one of the crucial healthcare challenges of our time (1–3). A systematic review by Anderson et al. in 2015 reported a prevalence of up to 34% in juveniles with obesity, being more common in males (4).

NAFLD is regarded as the manifestation of the metabolic syndrome in liver (5–7). Although the causative pathophysiological background of NAFLD in pediatric patients still needs further investigation, NAFLD has repeatedly been linked to obesity and insulin resistance (IR) as well as other comorbidities in adults as well as juveniles (8–11). Fang et al. (9) described a “multiple-hit hypothesis” leading to NAFLD, in which fat accumulation and consequently systemic and specifically hepatic insulin resistance play a major role.

Up to date, various mathematically calculable indices of hepatic IR have been developed, among which some indices can be obtained from just a single fasting blood draw (such as the homeostatic assessment index, HOMA-IR), whereas for other indices multiple blood draws are required, possibly representing a more “dynamic” state (such as the Hepatic Insulin Resistance Index, HIRI) (11–18). Recently, Bedogni et al. developed fatty liver prediction models based on Body-Mass-Index (BMI) or waist circumference, alanine aminotransferase, Homeostatic Model Assessment, triglycerides and uric acid to diagnose fatty liver in children with obesity (19). Previously, we developed a simple and inexpensive index, called the “Single Point Insulin Sensitivity Estimator” (SPISE), validated against the gold standard for assessing insulin sensitivity, the hyperinsulinemic-euglycemic clamp test (17), in an adult as well as in a juvenile cohort (20). This index consists of anthropometric as well as laboratory parameters, which enables clinicians to easily diagnose insulin resistance in pediatric patients, whose care calls for non-invasive and broadly accessible tools.

Based upon these considerations, the current study aimed to compare the performance of SPISE to established indices of hepatic IR in pediatric NAFLD-patients with obesity.

## Material And Methods

### Study Population and Design

Patients (n = 99) were recruited in an obesity specialist clinic in Salzburg (Austria) as part of the BETA JUDO study (BETA cell function in JUvenile Diabetes and Obesity, FP7-HEALTH-2011-two-stage, project number: 279153). Inclusion criteria were age 10-18 years and overweight or obesity according to the WHO criteria (BMI-SDS > 1). Written informed consent was obtained by all caregivers if patients were under the age of 18 years. Exclusion criteria were lack of consent or any chronic liver disease (such as hepatitis B and C). Patients did not report any alcohol intake. Height and bodymass were assessed by means of a standardized, calibrated scale (Seca, Hamburg, Germany). BMI and BMI-SDS were calculated according to the WHO 2006-2007 reference population (21). Waist circumference (cm), hip circumference (cm) and neck circumference (cm) were measured using a flexible tape. Blood pressure was measured twice, using a standardized clinical aneroid sphygmomanometer (Philips patient monitor MP30, Amsterdam, The Netherlands), and the mean value was recorded. Puberty staging was done according to Tanner (by a physician) and all subjects included into this study were staged as pubertal (Tanner II-IV).

### Blood Sampling and Biochemical Analyses

After an overnight fast, all patients underwent a standardized oral glucose tolerance test (OGTT, 1.75 g glucose/kg body mass) over 180 minutes as previously described (22, 23). OGTT was performed according to standard procedures by setting an intravenous line in an antecubital vein and subsequent blood draws were performed *via* this line at nine different time points after glucose challenge.

Uric acid, triglycerides, HDL cholesterol, total cholesterol and liver transaminases were measured using an enzymatic photometric test (Modular Analytics System, P-Modul 917, Roche Diagnostics, Vienna, Austria). The evaluation of LDL cholesterol also required an enzymatic photometric test using Integra Manual by Roche Diagnostics. Apolipoprotein (A2) and apolipoprotein (B) as well as high-sensitive CRP were examined by an immunologic turbidimetric test (COBAS- Integra, Roche Diagnostics, Vienna, Austria) and interleukin 6 by an enzyme-linked immunosorbent assay (Modular Analytics System, E-Modul by Roche Diagnostics). Leptin and adiponectin were determined manually using ELISA (Human Leptin ELISA, Biovendor, Brno, Czech Republic; Quantikine ELISA, Human Total Adiponectin/Acrp30 Immunoassay, R&D Systems, Inc., Minneapolis, MN, USA). HbA1c was measured by reversed-phase chromatography and lipoprotein (a) by a turbidimetric test (COBAS- Integra, Roche Diagnostics, Vienna, Austria). Samples underwent immediate centrifugation at 2500g for 10 minutes at 4°C, subsequently aliquoted and frozen at -80°C. Plasma was consecutively used for analyses of insulin, proinsulin and C-peptide in the central lab in Uppsala. Single-plex ELISA-kits for each analyte were used (Mercodia AB^®^, Uppsala, Sweden).

### Hyperinsulinemic Clamp Test

Euglycemic-hyperinsulinemic clamp tests were used to determine insulin sensitivity within an interval of maximally 3 to 4 weeks after the OGTT and after an overnight fast. The euglycemic clamp glucose target was calculated as the mean value of 3 fasting plasma glucose measurements. The glucose clamp target was set to 80 mg/dL (4.44 mmol/L) in case of a value above 80 mg/dL, and in case of a value above 100 mg/dL (5.55 mmol/L) the clamp goal was 100 mg/dL. Clamp tests were performed for 120 min, with primed-continuous regular insulin infusion [40 mU insulin * min^-1^ * (m^2^ total body surface area)^-1^]. Blood samples for the determination of serum insulin and C-peptide were drawn at 0 and 120 minutes and the glucose disposal rate (M-value; milligrams per kilogram per minute) was calculated (20, 24–26).

### Magnetic Resonance Imaging (MRI)

MRI-examinations were performed to determine liver fat content (LFC) and volumes of abdominal visceral adipose tissue (VAT) and subcutaneous adipose tissue (SAT) as previously described (23). All exams were performed using 1.5T clinical MRI-systems from Philips Medical System (Amsterdam, The Netherlands) after a light meal and in close proximity to the OGTT. Water-fat imaging techniques were used throughout. The scans were done over 16 cm along the craniocaudal axis and centered on the L1 vertebra. The adipose tissue volumes were determined using a fully automated segmentation method that uses a filtering technique to separate VAT from SAT. Liver fat image reconstruction was done by a multi-resolution version of a method that employs a whole-image optimization approach (27). A single operator trained by an experienced radiologist performed the measurements by manual segmentation in the axial slices of the water images using the software ImageJ (version 1.42q, http://rsbweb.nih.gov/ij/).

### Definition of NAFLD

Patients with NAFLD had a liver fat content >5%, as measured by MRI. This has previously been described and a close relation between histopathological changes and liver fat fraction in MR-imaging has been promoted by various groups (28–34).

### Definition of Hepatic Insulin Resistance

Hepatic insulin resistance was analyzed using Homeostatic Model Assessment or HOMA-IR [22.5/fasting insulin * fasting glucose], the Single Point Insulin Sensitivity Estimator or SPISE [600 * HDL-cholesterol^0,185^/(Triglycerides^0,2^ × BMI^1,338^)], and the Hepatic Insulin Resistance Index [(Glucose AUC_0–30_) x (Insulin AUC_0–30_)] (11–18, 20).

### Statistical Analysis

Descriptive data analysis showed results with mean and standard deviation for continuous variables and number and percentages for categorical variables. Pearson correlation was calculated to show linear dependencies. Receiver operating characteristic (ROC) curves were calculated showing sensitivity (true positive rate) and 1 – specificity (false positive rate) for each threshold of the indicator variable. Graphical representation was combined with area under the curve (AUC) as numerical measure indicating the classification quality. AUC was calculated using trapezoidal rule. The null hypothesis H0: AUC = 0.5 (indicating random classification) was tested using the Wilcoxon Mann Whitney test (H1: AUC > 0.5). Cutoff levels for SPISE were obtained using the maximum of the Youden index (= sensitivity + specificity – 1) (35). All results are presented along with 95%- confidence intervals. Significance was assumed at p<0.05. Due to exploratory analysis, p-values are not corrected for multiple testing. All calculations were done with R (The R Project, Version 3.6.0, Linz, Austria).

## Results

### Descriptive Data of All Patients

A total of 99 patients with obesity were included into this study (male: 59.4%, female: 40.4%). The age of patients was 13.5 ± 2.0 years. Further group characteristics on anthropometric and biochemical parameters are shown in **Table 1**.

**Table 1:** Descriptive data of all patients (n = 99^#^).

	Mean	± SD
Age (years)	13.5	2.0
Gender	male: 59 (59.6%) female: 40 (40.4%)	
**Anthropometric data**		
Body mass (kg)	86.6	21.0
Height (cm)	164.2	11.2
BMI (kg/m^2^)	31.8	5.6
BMI-SDS	2.8	0.6
SBMI (kg/m^2^)	34.9	4.2
Waist circumference (cm)	102.4	12.9
Waist/ Hip ratio	1.0	0.1
Systolic blood pressure (mmHg)	121.6	12.0
**MRI data**		
MRI liver fat content (%)	10.1	10.5
MRI VAT volume (cm^3^)	1474.2	558.5
MRI SAT volume (cm^3^)	6412.5	2209.7
MRI DSAT volume (cm^3^)	3095.1	1228.4
MRI SSAT volume (cm^3^)	3085.0	1151.5
**Laboratory data**		
HbA1c (mmol/mol)	35.0	2.4
Total cholesterol (mmol/L)	4.2	0.8
LDL cholesterol (mmol/L)	2.3	0.7
HDL cholesterol (mmol/L)	1.3	0.4
Triglycerides (mmol/L)	1.2	0.6
AST (μkat/L)	0.5	0.4
ALT (μkat/L)	0.6	0.7
GGT (μkat/L)	0.4	0.3
Uric acid (μmol/L)	351.1	84.0
Adiponectin (μg/mL)	7.7	3.3
Leptin (ng/mL)	36.2	23.9
hs-CrP (mg/L)	3.9	4.3
IL-6 (pg/mL)	7.4	2.4
TNF-alpha (pg/mL)	8.3	1.9
**OGTT data**		
OGTT fasting glucose (mmol/L)	4.8	0.6
OGTT 120 min. glucose (mmol/L)	6.3	1.4
OGTT fasting insulin (pmol/L)	120.2	64.9
**Parameters of insulin resistence**		
SPISE	5.2	1.4
HOMA-IR	3.6	2.0
HIRI	55928.4	33615.0

^#^n = 99, except for n = 98 for waist circumference, waist/hip ratio, systolic blood pressure, HbA1c, uric acid, hs-CrP; n = 103 for MRI VAT, MRI SAT, OGTT fasting glucose; n = 96 for MRI DSAT, MRI SSAT; n = 95 for AST; n = 90 for IL-6; n = 89 for TNF-alpha; n = 87 for adiponectin; n = 72 for OGTT fasting insulin; n = 71 for HOMA-IR; n = 72 for leptin; n = 64 for HIRI.

Data are expressed as mean ± standard deviation. All subjects were staged as “pubertal” according to Tanner staging (II-IV).

SD, standard deviation; BMI, body mass index; SDS, standard deviation score; SBMI, smart body mass index; MRI, magnetic resonance imaging; VAT, visceral adipose tissue; SAT, subcutaneous adipose tissue; DSAT, deep subcutaneous adipose tissue; SSAT, superficial subcutaneous adipose tissue; HbA1c, hemoglobin A1c; LDL, low density lipoprotein; HDL, high density lipoprotein; AST, aspartate transaminase; ALT, alanine transaminase; GGT, gamma-glutamyl transferase; hs-CrP, high-sensitivity C-reactive Protein; IL-6, Interleukin 6; TNF, tumor necrosis factor; OGTT, oral glucose tolerance test; min., minutes; SPISE, Single Point Insulin Sensitivity Estimator; HOMA-IR, homeostatic Model Assessment for Insulin Resistance; HIRI, Hepatic Insulin Resistance Index.

### Descriptive Data of NAFLD and Non-NAFLD Groups

Patients were further categorized into the ones with and the ones without NAFLD, as defined by MRI-measured liver fat content. They were separated into male and female groups with 41 male and 16 female NAFLD patients as well as 18 male and 24 female non-NAFLD patients. Ages ranged between 12.7 ± 2.2 and 14.3 ± 2.4 years respectively (details see **Table 2**). Liver fat content was highest in the male NAFLD group (15.9 ± 11.9% {confidence interval [CI]: 12.1 - 19.6%}) in comparison to all non-NAFLD patients (male non-NAFLD 3.0 ± 1.0%, P<0.001 {95% confidence interval [CI]: 2.5, 3.5%}; female non-NAFLD 3.1 ± 0.9%, P<0.001 {95% confidence interval [CI]: 2.7, 3.4%}). Liver fat content in female NAFLD patients was 13.5 ± 9.7% (P<0.600 {95% confidence interval [CI]: 8.3, 18.7%}). Further details of anthropometric and biochemical parameters are shown in **Table 2**. SPISE was lower in NAFLD (male: 4.8 ± 1.2, female: 4.5 ± 1.1) than in non-NAFLD group (male 6.0 ± 1.6, female 5.6 ± 1.5; *P*< 0.05 {95% confidence interval [CI]: male NAFLD 4.5, 5.2; male non-NAFLD 5.2, 6.8; female NAFLD 4.0, 5.1; female non-NAFLD 5.0, 6.2}).

**Table 2:** Descriptive data of NAFLD (male n = 41^§^, female n = 16^+^) and non-NAFLD patients (male n = 18^#^, female n = 24^$^). NAFLD was defined as liver fat content > 5% according to MRI).

	Male		Female	
	NAFLD	non-NAFLD	NAFLD	non-NAFLD
**Anthropometric data**				
Age (years)	13.6 ± 1.9	13.6 ± 1.7	14.3 ± 2.4	12.7 ± 2.2
Body mass (kg)	90.8 ± 21.0	88.4 ± 21.5	88.9 ± 17.3	76.3 ± 20.6
Height (cm)	166.0 ± 11.9	171.5 ± 8.1	161.1 ± 7.5	157.8 ± 10.1
BMI (kg/m^2^)	32.7 ± 5.0	29.7 ± 4.8	34.3 ± 6.8	30.3 ± 5.8
BMI-SDS	3.0 ± 0.5	2.6 ± 0.6	2.9 ± 0.8	2.6 ± 0.6
SBMI (kg/m^2^)	35.8 ± 3.5	33.2 ± 3.3	36.1 ± 5.5	34.0 ± 4.5
Waist circumference (cm)	106.7 ± 11.4	99.9 ± 14.3	102.3 ± 10.7	96.9 ± 13.8
Waist/ Hip ratio	**1.0 ± 0.1** [0.97, 1.01]	**0.9 ± 0.1** [0.89, 0.96]	0.9 ± 0.1	0.9 ± 0.1
Systolic blood pressure (mmHg)	123.6 ± 13.2	122.0 ± 11.9	120.0 ± 9.3	119.0 ± 11.5
**MRI data**				
MRI liver fat content (%)	**15.9 ± 11.9** [12.12, 19.64]	**3.0 ± 1.0** [2.54, 3.49]	**13.5 ± 9.7** [8.35, 18.71]	**3.1 ± 0.9** [2.71, 3.44]
MRI VAT volume (cm^3^)	**1722.3 ± 654.1** [1515.83, 1928.73]	**1244.4 ± 337.8** [1076.43, 1412.44]	**1572.1 ± 370.2** [1374.82, 1769.35]	**1158.3 ± 388.6** [994.16, 1322.34]
MRI SAT volume (cm^3^)	6601.5 ± 2021.3	5518.4 ± 2397.2	7658.2 ± 2134.6	5900.1 ± 2116.0
MRI DSAT volume (cm^3^)	3256.9 ± 1196.3	2589.8 ± 1303.4	3660.7 ± 886.3	2874.5 ± 1271.1
MRI SSAT volume (cm^3^)	3134.7 ± 950.2	2628.8 ± 1084.2	3821.2 ± 1636.6	2914.6 ± 1017.8
**Laboratory data**				
HbA1c (mmol/mol)	35.3 ± 3.0	35.4 ± 2.1	34.8 ± 1.8	34.5 ± 1.8
Total cholesterol (mmol/L)	4.2 ± 0.9	4.0 ± 0.8	4.2 ± 0.7	4.3 ± 0.6
LDL cholesterol (mmol/L)	2.4 ± 0.8	2.1 ± 0.5	2.2 ± 0.5	2.4 ± 0.6
HDL cholesterol (mmol/L)	1.3 ± 0.2	1.5 ± 0.5	1.4 ± 0.3	1.4 ± 0.4
Triglycerides (mmol/L)	1.2 ± 0.7	0.9 ± 0.5	1.3 ± 0.4	1.1 ± 0.5
AST (μkat/L)	0.7 ± 0.5	0.5 ± 0.1	0.4 ± 0.1	0.4 ± 0.2
ALT (μkat/L)	**0.9 ± 1.0** [0.56, 1.16]	**0.4 ± 0.2** [0.35, 0.54]	0.4 ± 0.1	0.3 ± 0.1
GGT (μkat/L)	0.5 ± 0.4	0.3 ± 0.1	0.3 ± 0.1	0.3 ± 0.1
Uric acid (μmol/L)	360.8 ± 99.9	384.5 ± 84.3	339.8 ± 48.7	317.2 ± 61.0
Adiponectin (μg/mL)	6.6 ± 2.4	9.2 ± 4.6	7.5 ± 3.1	8.5 ± 3.2
Leptin (ng/mL)	32.6 ± 18.4	26.3 ± 33.3	45.3 ± 19.0	43.7 ± 24.9
hs-CrP (mg/L)	4.3 ± 4.9	3.2 ± 3.5	4.5 ± 3.8	3.1 ± 4.0
IL-6 (pg/mL)	7.9 ± 3.7	7.0 ± 0.1	7.2 ± 0.6	7.2 ± 1.0
TNF-alpha (pg/mL)	8.6 ± 1.4	8.1 ± 2.0	7.4 ± 2.1	8.5 ± 2.2
**OGTT data**				
OGTT fasting glucose (mmol/L)	4.9 ± 0.6	4.9 ± 0.6	4.7 ± 0.7	4.8 ± 0.6
OGTT 120 min. glucose (mmol/L)	6.6 ± 1.4	6.0 ± 1.4	6.1 ± 1.7	6.4 ± 1.2
OGTT fasting insulin (pmol/L)	137.7 ± 74.9	94.5 ± 48.0	131.2 ± 79.4	100.2 ± 31.0
**Parameters of insulin resistance**				
SPISE	4.8 ± 1.2	6.0 ± 1.6	4.5 ± 1.1	5.6 ± 1.5
HOMA-IR	4.2 ± 2.2	2.9 ± 1.6	4.0 ± 2.6	2.9 ± 0.9
HIRI	56543.0 ± 31614.9	54967.8 ± 32057.1	66954.9 ± 40217.0	48134.5 ± 35071.6

Data are expressed as mean ± standard deviation. All subjects were staged as “pubertal” according to Tanner staging (II-IV).

^§^n = 41 except of n = 40 for waist circumference, waist/hip ratio, systolic blood pressure, HbA1c, AST, uric acid, OGTT fasting glucose, VAT, SAT, DSAT, SSAT; n = 36 for IL-6, TNF-alpha; n = 33 for adiponectin; n = 32 for OGTT fasting insulin; n = 31 for HOMA-IR; n = 30 for leptin; n = 28 for HIRI.

^+^n = 16 except of n = 15 for OGTT fasting glucose; n = 14 for AST, DSAT, SSAT; n = 13 for leptin; n = 10 for OGTT fasting insulin, HOMA-IR, HIRI.

^#^n = 18 except of n = 17 for adiponectin, IL-6, TNF-alpha, VAT, SAT; n = 13 for leptin; n = 12 for OGTT fasting insulin, HOMA-IR; n = 11 for HIRI.

^$^n = 24 except of n = 23 for AST, hs-CrP; n = 21 for adiponectin, IL-6; n = 20 for TNF-alpha; n = 18 for OGTT fasting insulin, HOMA-IR; n = 16 for leptin; n = 15 for HIRI.

NAFLD, non-alcoholic fatty liver disease; MRI, magnetic resonance imaging; SD, standard deviation; BMI, body mass index; SDS, standard deviation score; SBMI, smart body mass index; VAT, visceral adipose tissue; SAT, subcutaneous adipose tissue; DSAT, deep subcutaneous adipose tissue; SSAT, superficial subcutaneous adipose tissue; HbA1c, hemoglobin A1c; LDL, low density lipoprotein; HDL, high density lipoprotein; AST, aspartate transaminase; ALT, alanine transaminase; GGT, gamma-glutamyl transferase; hs-CrP, high-sensitivity C-reactive Protein; IL-6, Interleukin 6; TNF, tumor necrosis factor; OGTT, oral glucose tolerance test; min., minutes; SPISE, Single Point Insulin Sensitivity Estimator; HOMA-IR, homeostatic Model Assessment for Insulin Resistance; HIRI, Hepatic Insulin Resistance Index.

Confidence intervals were calculated and significant differences between NAFLD and non-NAFLD groups were marked in bold letters and the 95% confidence interval (CI) added in brackets.

**Figure 1:**
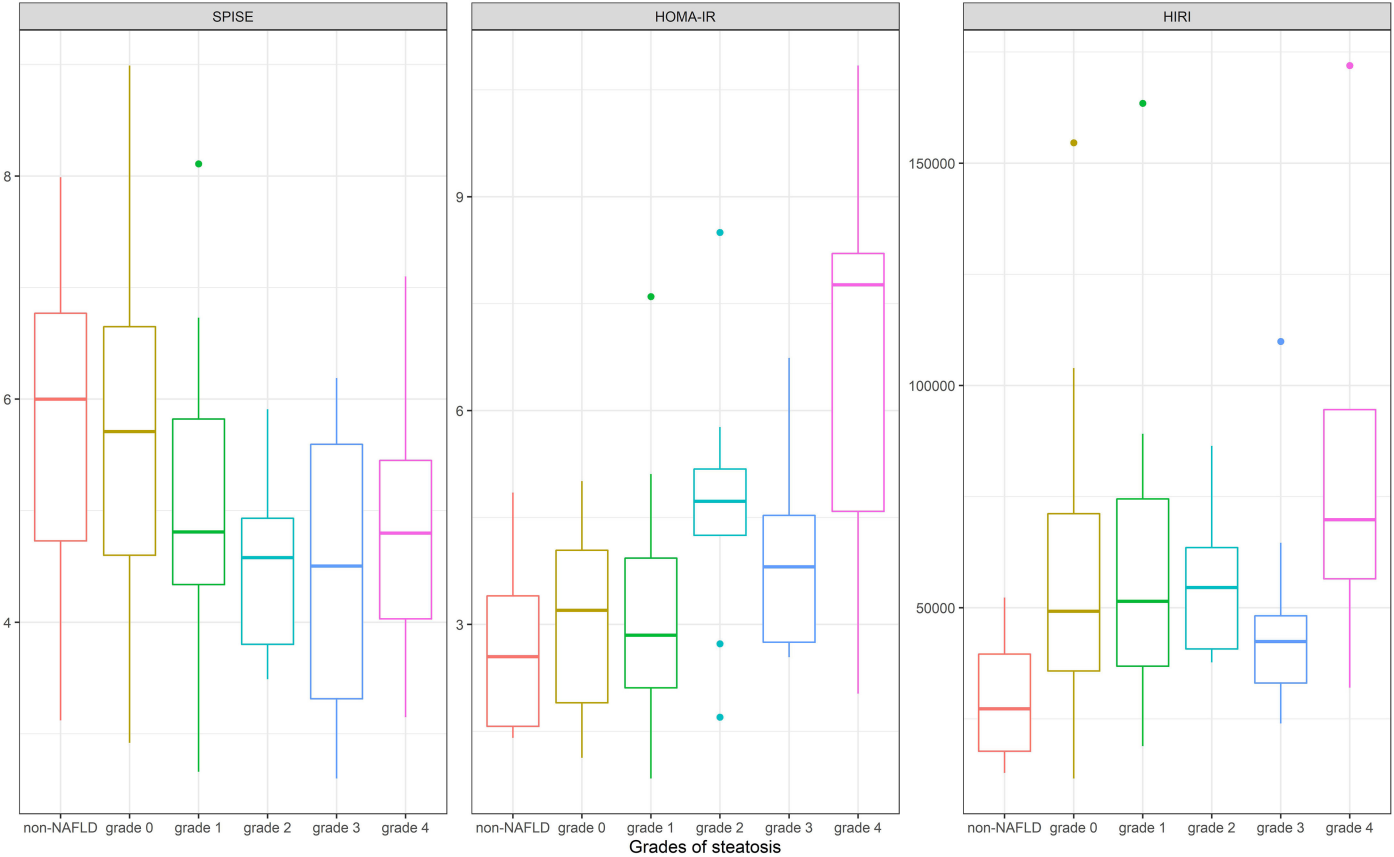
Comparison of the performance of SPISE, HOMA-IR and HIRI according to different steatosis grades [non-NAFLD: liver fat content (LFC) < 2.6%; grade 0: LFC 2.6 - ≤5%; grade 1: LFC >5 - ≤9.2%; grade 2: LFC >9.2 - ≤15.1%; grade 3 : LFC >15.1 - ≤26.8%; grade 4: LFC >26.8%]. SPISE, Single Point Insulin Sensitivity Estimator; HOMA-IR, homeostatic Model Assessment for Insulin Resistance; HIRI, Hepatic Insulin Resistence Index; NAFLD, Non-alcoholic fatty liver disease; LFC, Liver fat content.

In a subgroup analysis in **Table 3**, considering NAFLD according to grade of steatosis as measured *via* MRI, SPISE was significantly lower in patients with higher NAFLD grades respectively more steatosis (non-NAFLD compared to NAFLD grades 1-4: P<0.001). **Figure 1** compared the performance of SPISE, HOMA-IR and HIRI in different steatosis grades. SPISE as well as HOMA-IR and HIRI were not significantly different in higher steatosis grades (2-4).

**Table 3:** Descriptive data of NAFLD and non-NAFLD subjects according to grades of steatosis: non-NAFLD^#^: liver fat content (LFC) < 2.6%; grade 0^+^: LFC 2.6 - ≤5%; grade 1^§^: LFC >5 - ≤9.2%; grade 2^%^: LFC >9.2 - ≤15.1%; grade 3 ^o^: LFC >15.1 - ≤26.8%; grade 4^♦^: LFC >26.8%. Non-NAFLD in this study was defined as a LFC ≤5%, therefore it comprises of following groups in the table: non-NAFLD and NAFLD grade 0.

	non-NAFLD	NAFLD grade 0	NAFLD grade 1	NAFLD grade 2	NAFLD grade 3	NAFLD grade 4
**Anthropometric data**						
Age (years)	13.1 ± 2.1	13.1 ± 2.0	14.4 ± 2.1	13.5 ± 1.7	13.7 ± 2.2	12.9 ± 1.8
Body mass (kg)	82.6 ± 26.3	81.0 ± 19.6	90.9 ± 19.0	88.3 ± 17.1	92.8 ± 23.0	88.0 ± 24.4
Height (cm)	165.2 ± 12.9	162.9 ± 11.0	166.5 ± 9.6	160.7 ± 12.3	165.1 ± 11.1	164.1 ± 12.8
BMI (kg/m^2^)	29.6 ± 5.7	30.2 ± 5.3	32.8 ± 6.5	34.1 ± 4.8	33.7 ± 5.1	32.3 ± 4.5
BMI-SDS	2.6 ± 0.6	**2.6 ± 0.6** [2.42, 2.87]	2.8 ± 0.7	**3.1 ± 0.4** [2.88, 3.73]	3.1 ± 0.5	3.0 ± 0.6
SBMI (kg/m^2^)	33.1 ± 3.7	33.9 ± 4.2	35.1 ± 5.0	36.6 ± 2,9	36.6 ± 3.3	36.1 ± 3.9
Waist circumference (cm)	99.9 ± 17.1	97.5 ± 12.5	104.6 ± 12.6	107.0 ± 9.8	105.1 ± 10.9	106.3 ± 11.8
Waist/ Hip ratio	1.0 ± 0.1	**0.9 ± 0.1** [0.89, 0.94]	1.0 ± 0.1	**1.0 ± 0.0** [0.95, 1.00]	1.0 ± 0.1	**1.0 ± 0.1** [0.96, 1.05]
Systolic blood pressure (mmHg)	120.1 ± 11.3	120.3 ± 12.0	121.1 ± 10.0	120.2 ± 12.1	124.5 ± 13.5	126.9 ± 16.4
**MRI data**						
MRI VAT volume (cm^3^)	**1114.7 ± 350.4** [902.98, 1326.50]	**1226.8 ± 374.0** [1084.53, 1369.03]	1465.5 ± 419.3	**1801.8 ± 512.2** [1476.33, 2127.18]	1763.3 ± 800.5	**2011.5 ± 629.6** [1527.55, 2495.49]
MRI SAT volume (cm^3^)	5769.0 ± 2911.1	5730.6 ± 1919.2	7038.8 ± 2428.1	7082.6 ± 1752.9	6666.2 ± 1742.7	6584.3 ± 2246.2
MRI DSAT volume (cm^3^)	2540.8 ± 1491.8	2847.4 ± 1185.1	3270.2 ± 1159.4	3647.4 ± 1090.0	3262.7 ± 942.6	3341.3 ± 1528.3
MRI SSAT volume (cm^3^)	2648.6 ± 1341.0	2856.5 ± 900.1	3521.3 ± 1478.7	3156.6 ± 921.1	3170.1 ± 865.0	3139.4 ± 1118.0
MRI liver fat content (%)	**1.9 ± 0.4** [1.70, 2.19]	**3.6 ± 0.5** [3.35, 3.74]	**6.3 ± 1.1** [5.81, 6.76]	**11.8 ± 1.4** [10.87, 12.69]	**20.2 ± 3.1** [18.16, 22.14]	**37.1 ± 6.7** [31.94, 42.16]
**Laboratory data**						
HbA1c (mmol/mol)	**33.5 ± 1.6** [32.56, 34.51]	**35.5 ± 1.9** [34.77, 36.19]	35.0 ± 2.2	35.9 ± 2.4	34.9 ± 4.2	34.6 ± 2.2
Total cholesterol (mmol/L)	3.9 ± 0.8	4.3 ± 0.6	4.2 ± 0.9	4.2 ± 0.6	4.4 ± 0.8	3.9 ± 1.1
LDL cholesterol (mmol/L)	2.3 ± 0.7	2.3 ± 0.6	2.4 ± 0.8	2.4 ± 0.5	2.5 ± 0.8	2.0 ± 0.9
HDL cholesterol (mmol/L)	1.2 ± 0.2	1.5 ± 0.5	1.3 ± 0.2	1.3 ± 0.3	1.2 ± 0.3	1.2 ± 0.2
Triglycerides (mmol/L)	0.9 ± 0.3	1.1 ± 0.6	1.1 ± 0.5	1.1 ± 0.5	1.4 ± 0.7	1.5 ± 1.0
AST (μkat/L)	0.5 ± 0.2	0.4 ± 0.1	0.4 ± 0.1	0.5 ± 0.1	0.7 ± 0.5	1.0 ± 0.8
ALT (μkat/L)	**0.3 ± 0.1** [0.29, 0.40]	0.4 ± 0.2	**0.4 ± 0.1** [0.34, 0.42]	0.5 ± 0.2	**0.9 ± 0.8** [0.43, 1.41]	**1.7 ± 1.5** [0.57, 2.90]
GGT (μkat/L)	**0.2 ± 0.1** [0.21, 0.28]	0.3 ± 0.1	0.3 ± 0.1	**0.3 ± 0.1** [0.28, 0.40]	0.4 ± 0.2	**0.9 ± 0.8** [0.29, 1.51]
Uric acid (μmol/L)	357.9 ± 74.5	340.7 ± 81.0	338.6 ± 87.0	333.1 ± 87.1	380.0 ± 59.4	391.2 ± 117.2
Adiponectin (μg/mL)	7.7 ± 4.7	9.1 ± 3.6	7.3 ± 1.9	8.4 ± 3.9	5.5 ± 2.2	6.0 ± 2.9
Leptin (ng/mL)	22.1 ± 26.4	40.2 ± 30.0	34.9 ± 20.1	44.3 ± 15.0	35.0 ± 20.7	33.7 ± 21.9
hs-CrP (mg/L)	4.7 ± 5.4	2.4 ± 2.5	4.3 ± 4.3	5.5 ± 5.6	2.6 ± 2.0	5.4 ± 6.1
IL-6 (pg/mL)	7.1 ± 0.2	7.2 ± 0.9	7.6 ± 2.6	7.0 ± 0.1	7.0 ± 0.0	9.4 ± 6.6
TNF-alpha (pg/mL)	8.0 ± 1.7	8.4 ± 2.2	8.2 ± 1.6	8.1 ± 1.7	8.2 ± 2.1	8.5 ± 1.5
**OGTT data**						
OGTT fasting glucose (mmol/L)	4.9 ± 0.7	4.9 ± 0.5	4.6 ± 0.5	5.2 ± 0.9	4.8 ± 0.6	5.0 ± 0.5
OGTT 120 min. glucose (mmol/L)	5.9 ± 1.7	**6.4 ± 1.1** [5.21, 6.38]	5.8 ± 1.4	6.8 ± 1.5	6.4 ± 0.8	**7.8 ± 1.5** [6.64, 8.86]
OGTT fasting insulin (pmol/L)	**92.1 ± 46.9** [63.72, 120.45]	**100.4 ± 34.6** [87.28, 113.58]	**103.8 ± 55.1** [80.56, 127.07]	146.6 ± 62.0	135.9 ± 54.4	**217.8 ± 114.4** [129.87, 305.72]
**Parameters of insulin resistance**						
SPISE	5.9 ± 1.5	**5.7 ± 1.6** [5.14, 6.35]	5.0 ± 1.3	**4.5 ± 0.8** [4.03, 5.03]	4.5 ± 1.3	4.9 ± 1.2
HOMA-IR	**2.7 ± 1.2** [1.95, 3.40]	**3.0 ± 1.2** [2.57, 3.49]	**3.0 ± 1.7** [2.30, 3.72]	**4.7 ± 1.9** [3.49, 5.91]	3.9 ± 1.4	**6.7 ± 3.3** [4.19, 9.24]
HIRI	**29659.4 ± 15652.8** [20200.50, 39118.32]	**57435.4 ± 34798.1** [44198.85, 70671.86]	**59224.3 ± 35461.2** [44250.31, 74198.24]	**55862.6 ± 17165.9** [44955.87, 66769.28]	48151.8 ± 26004.5	**84979.5 ± 53611.3** [43770.26, 126188.80]

Data are expressed as mean ± standard deviation. All subjects were staged as “pubertal” according to Tanner staging (II-IV).

Each group contains male as well as female patients; detailed information on distribution is available on demand.

Exceptions in number of patients: ^#^n = 13, except for AST/MRI VAT/MRI SAT (n=12), adiponectin/IL-6/TNF-alpha/OGTT fasting insulin/HOMA-IR (n=9), leptin (n=7), HIRI (n=6); ^+^n = 29, except for hs-CrP/TNF-alpha (n=28), leptin (n=22), OGTT fasting insulin/HOMA-IR (n=21), HIRI (n=20); ^§^n = 24, except for HbA1c/adiponectin/DSAT/SSAT (n=23), leptin/OGTT fasting glucose (n=22), AST (n=21), OGTT fasting insulin (n=18), HOMA-IR (n=17), HIRI (n=16); ^%^n = 12, except for waist circumference/waist-hip-ratio/systolic blood pressure/uric acid (n=11), IL-6/TNF-alpha/OGTT fasting insulin/HOMA-IR (n=9), adiponectin/leptin/HIRI (n=8); ^o^n = 12, except for IL-6/TNF-alpha (n=11), adiponectin (n=10), OGTT fasting insulin/HOMA-IR/HIRI (n=9), leptin (n=7); ^♦^n = 9, except for adiponectin/IL-6/TNF-alpha/MRI VAT/MRI SAT (n=8), MRI DSAT/MRI SSAT (n=7), leptin/OGTT fasting insulin/HOMA-IR (n=6), HIRI (n=5).

NAFLD, non-alcoholic fatty liver disease; MRI, magnetic resonance imaging; SD, standard deviation; BMI, body mass index; SDS, standard deviation score; SBMI, smart body mass index; VAT, visceral adipose tissue; SAT, subcutaneous adipose tissue; DSAT, deep subcutaneous adipose tissue; SSAT, superficial subcutaneous adipose tissue; HbA1c, hemoglobin A1c; LDL, low density lipoprotein; HDL, high density lipoprotein; AST, aspartate transaminase; ALT, alanine transaminase; GGT, gamma-glutamyl transferase; hs-CrP, high-sensitivity C-reactive Protein; IL-6, Interleukin 6; TNF, tumor necrosis factor; OGTT, oral glucose tolerance test; min., minutes; SPISE, Single Point Insulin Sensitivity Estimator; HOMA-IR, homeostatic Model Assessment for Insulin Resistance; HIRI, Hepatic Insulin Resistance Index.

Confidence intervals were calculated and significant differences between groups were marked in bold letters and the 95% confidence interval (CI) is added in brackets.

### Comparison of Insulin Sensitivity Indices

We performed hyperinsulinemic clamp tests (n=17) and used calculated M-values as excepted means to estimate insulin sensitivity. As shown in **Table 4**, the correlation of M-values and SPISE (r = 0.49) is significantly greater than between M-values and HOMA-IR (r = 0.11) or, respectively, HIRI (r = -0.32).

**Table 4:** Pearson correlation coefficients (r) for the relation of M-value (100-120 min.) as derived from euglycemic clamp method and hepatic insulin resistance indices (n = 17).

	r-value	p-value	CI
**SPISE**	0.489	0.047*	[0.010 , 0.785]
**HOMA-IR**	-0.135	0.604	[-0.578 , 0.369]
**HIRI**	-0.323	0.362	[-0.792 , 0.385]

*p < 0.05.

CI, confidential interval; SPISE, Single Point Insulin Sensitivity Estimator; HOMA-IR, homeostatic Model Assessment for Insulin Resistance; HIRI, Hepatic Insulin Resistence Index.

### ROC-Curve Analysis and Optimal Cutoff Levels of SPISE in NAFLD Patients

**Figure 2:**
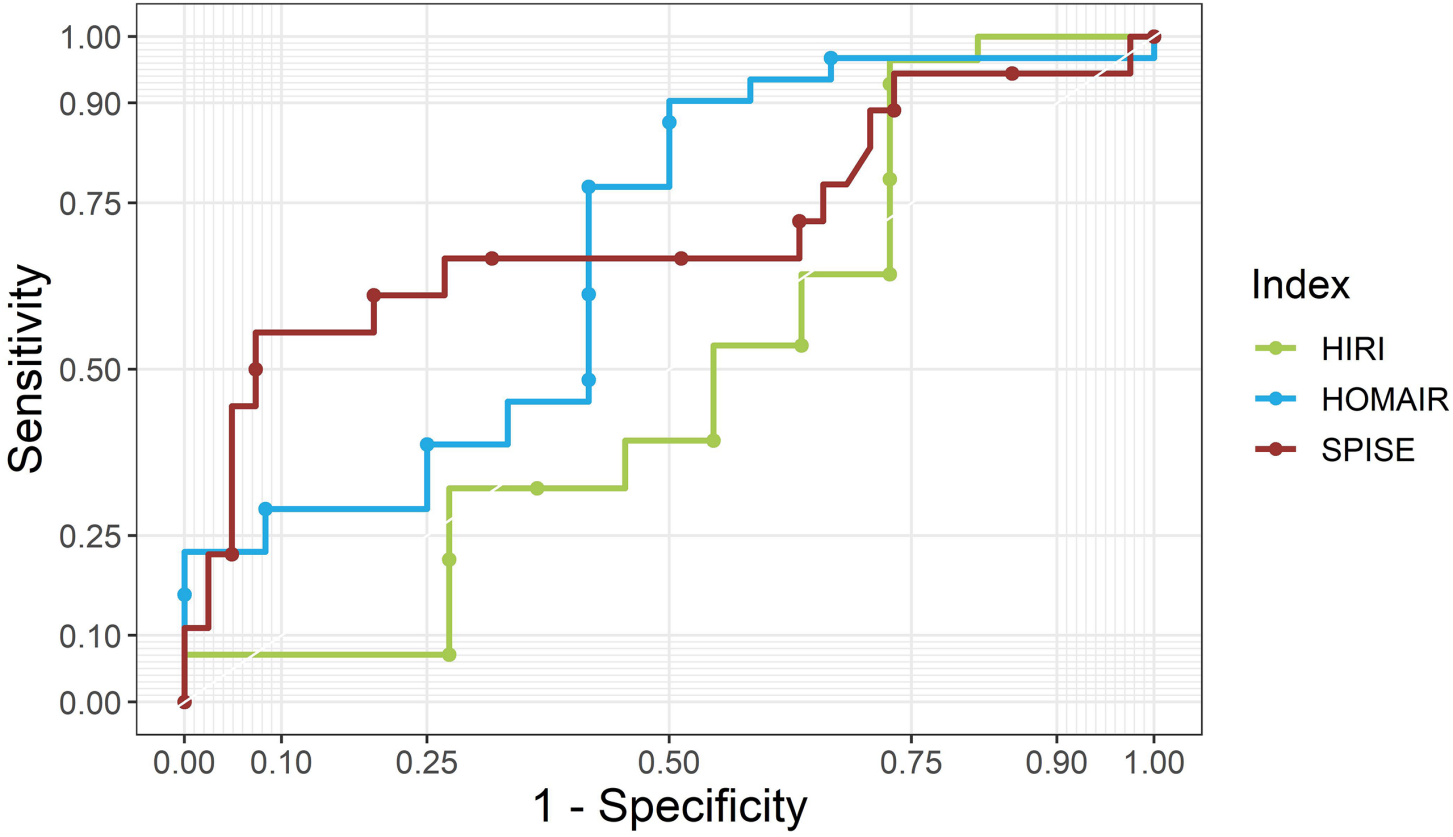
ROC curves for SPISE, HOMA-IR and HIRI for male patients. SPISE, Single Point Insulin Sensitivity Estimator; HOMA-IR, homeostatic Model Assessment for Insulin Resistance; HIRI, Hepatic Insulin Resistence Index.

**Figure 3:**
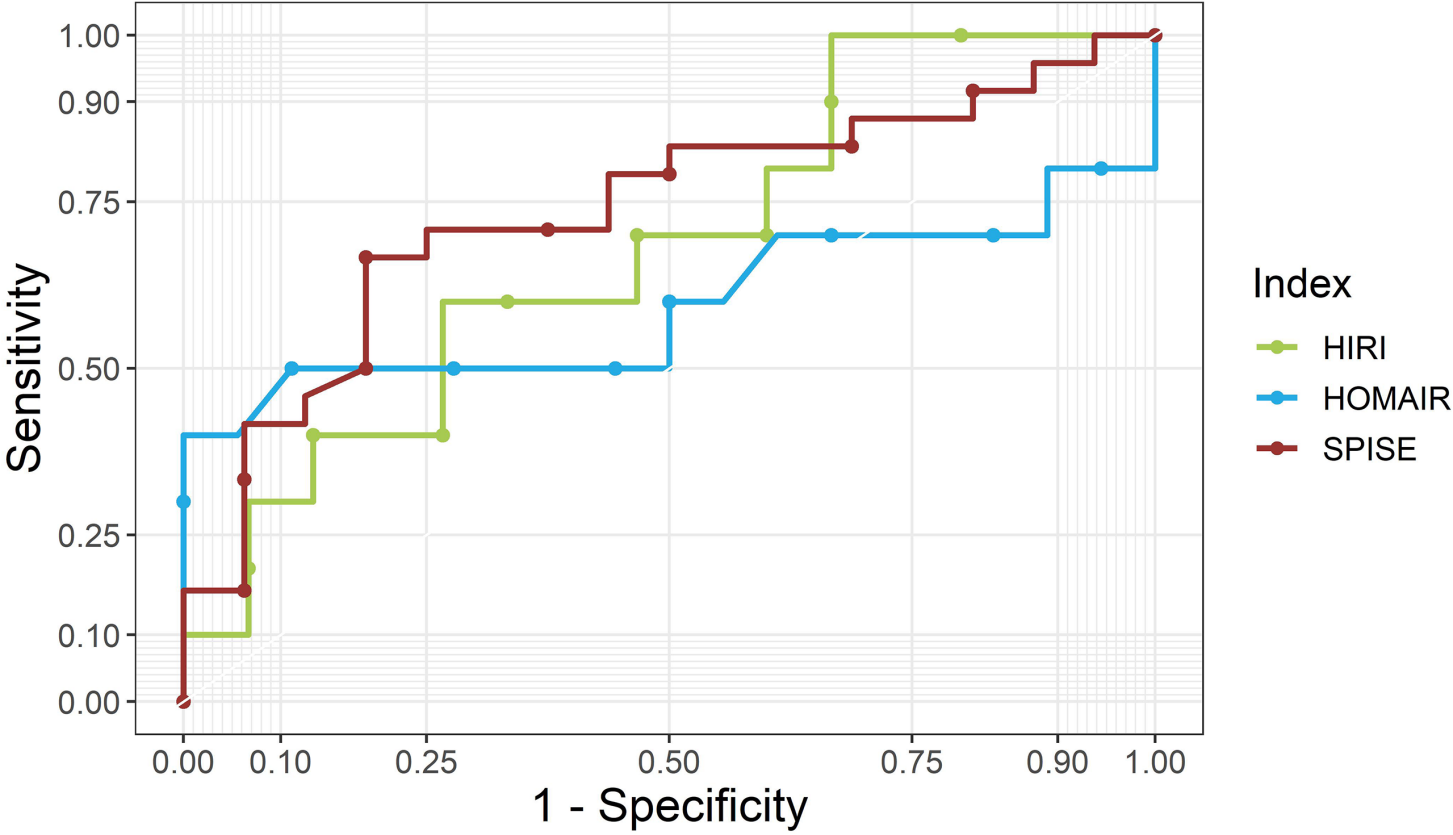
ROC curves for SPISE, HOMA-IR and HIRI for female patients. SPISE, Single Point Insulin Sensitivity Estimator; HOMA-IR, homeostatic Model Assessment for Insulin Resistance; HIRI, Hepatic Insulin Resistence Index.

Finally, we analyzed ROC-curves in male as well as female patients for SPISE, HOMA-IR, and HIRI (**Figures 2**, **3**). In male patients, ROC-curve showed AUC of 0.71 for SPISE (*P*=0.006, 95% CI: 0.54, 0.87), 0.68 for HOMA-IR (*P*=0.038, 95% CI: 0.48, 0.88), and 0.50 for HIRI (*P*=0.543, 95% CI: 0.27, 0.74). In female patients, ROC-AUC was 0.74 for SPISE (*P*=0.006, 95% CI: 0.58, 0.90), 0.59 for HOMA-IR (*P*=0.214, 95% CI: 0.32, 0.87), and 0.68 for HIRI (*P*=0.072, 95% CI: 0.46, 0.90). SPISE seemed to perform better in female patients compared to males (0.74 vs. 0.71 in males), but when comparing ROC-curves the difference was not significant (p=0.814).

The optimal cutoff level for SPISE between NAFLD and non-NAFLD patients was 5.18 overall (Youden index: 0.35; sensitivity 0.68%, specificity 0.67%). When looking at different NAFLD grades, as shown in **Table 3**, the optimal cutoff level was described as following: SPISE > 5.18 between non-NAFLD and NAFLD grades 1-2 (Youden index: 0.36; sensitivity 0.69%, specificity 0.67%), SPISE > 5.79 between non-NAFLD and NAFLD grades 3-4 (Youden index: 0.38; sensitivity 0.86%, specificity 0.52%) respectively.

## Discussion

The current study aimed to compare the performance of SPISE as an estimation of hepatic impaired insulin sensitivity in children and adolescents with obesity. The main finding of this study is that SPISE indicates hepatic IR in pediatric patients with sensitivity and specificity superior as compared to established indices of hepatic IR.

Childhood obesity and its comorbidities show a rising prevalence worldwide (1, 2), implicating that an early identification of these diseases is of utmost importance in order to achieve better patient outcomes. Among these comorbidities, NAFLD has been associated with a metabolic deterioration as early as during childhood (8–10). NAFLD predictive risk factors in childhood were demonstrated to include increased waist circumference, elevated waist-to-hip ratio, elevated total cholesterol, triglycerides, fasting insulin, HOMA-IR as well as elevated glucose and insulin concentration in an OGTT (36). Previously, the best independent predictive risk factor for diagnosing NAFLD in non-diabetic children with obesity was suggested to be fasting insulin >18.9 μIU/ml (36). However, fasting insulin and HOMA-IR levels vary considerably depending on the type of insulin assay (37, 38). Hence, multiple surrogate markers of IR have formerly emerged (20, 39, 40).

Among these, the SPISE was developed as an easy and affordable tool for the evaluation of whole-body insulin sensitivity, which is comparable to clamp-derived M-value in sensitivity as well as specificity (19). Several studies have evaluated the SPISE in adult as well as juvenile populations (20, 41–46). Correa-Burrows et al. assessed SPISE for its validity in diagnosing cardiometabolic risks, namely IR and metabolic syndrome, in post-pubertal Hispanic adolescents. SPISE was found to be accurate for the prediction of IR in both groups, with cutoff values of 5.0 (males) and 6.0 (females) indicating IR (41). Similarly, a cutoff value of 5.82 for prediction of IR in metabolic syndrome was determined by Dudi et al. in a north Indian adult population. SPISE was thereby shown to discriminate well between cases and controls (42). More recently, a study analysed data from 909 Italian children with overweight and obesity and normal weight controls undergoing metabolic evaluations. Two-hundred children who were overweight or obese were assessed longitudinally for on average of 6.5 years (range 3.5-10). At follow-up, lower basal SPISE strongly predicted the development of abnormal glucose metabolism (AUROC curve: 0.83 [0.72-0.94] regardless of age, sex, fasting/120 mins glucose and insulin at baseline (46). Of interest, SPISE-IR (=10/SPISE) was also a predictor of coronary heart disease and type 2 diabetes in a group of elderly Swedish men (44).

To the best of our knowledge, SPISE has so far not been assessed in children and adolescents with NAFLD. Our results are perfectly in line with data from the Yale Pediatric NAFLD cohort showing that intrahepatic lipid accumulation is associated with reduced insulin clearance and hepatic insulin sensitivity in youths with obesity, irrespective of their ethnic background (11).

Insulin resistance was shown to be indicative of histological severity of liver disease in adults with obesity (47, 48). Additionally, HOMA-IR was an independent predictor of advanced liver fibrosis in nondiabetic Japanese adults with NAFLD (49). Recently, Bedogni et al. developed two multivariable models, using single anthropometric as well as laboratory parameters (BMI or waist circumference, ALT, HOMA-IR, triglycerides and uric acid) (19). Both models were demonstrated to identify fatty liver, as diagnosed *via* ultrasonography (19). However, SPISE may offer an easier and therefore more accessible identification of patients with hepatic insulin resistance. Additionally, a radiologic diagnosis *via* MRI allowed us a much more accurate assessment of liver fat content compared to ultrasonography (28–34, 50).

As described before, SPISE is based on BMI, fasting HDL-cholesterol and triglyceride levels. Hepatic lipid accumulation is closely related to the development of IR (51). Elevated ceramide concentrations, together with their significant correlation with IR parameters in pediatric patients with obesity, were suggested to be associated with molecular pathways involved in insulin signaling impairment strongly linked to the pathogenesis of NAFLD (52). In addition, hepatic expression of genes associated with IR may drive NAFLD development and progression. Thus, genes which can promote intrahepatic fat accumulation, dysregulation of the lipid metabolism, lipotoxicity, and activation of cell survival pathways including activation of cell proliferation and differentiation pathways, were shown to allow classification of adult NASH (Nonalcoholic steatohepatitis)-with-fibrosis patients separately from mild-NAFL (nonalcoholic fatty liver) and NASH patients (53). In agreement with this, TG/HDL-C (triglyceride/HDL-cholesterol) ratio was reported to be useful to identify children and adolescents at high risk of NAFLD (54). This is also in accordance with data demonstrating that the fasting triglyceride-to glucose index was linked to increasing severity of hepatic steatosis and the presence of liver fibrosis in adults with NAFLD and more closely related to NAFLD and liver fibrosis compared to HOMA-IR after adjustment for confounding factors (55).

NAFLD is an exclusion diagnosis and can progress (NASH and fibrosis) if undiagnosed and untreated. A uniform international consent for screening for NAFLD in juvenile obesity does not exist. AASLD Guidance does not recommend screening for NAFLD in children with obesity due to “paucity of evidence” (56). In contrast, NASPGHAN advocates screening by alanine aminotransferase (ALT), but does not recommend ultrasound (US) due to low sensitivity in all children with overweight and obesity and additional risk factors at age 9-11 years (50). Both methods combined seem favorable as ALT might be normal or slightly elevated and US sensitivity diminishes in children where hepatic fat accumulation remains below 30% (57). Up to now, the gold standard in diagnosing fibrosis is liver biopsy, which nevertheless resembles an invasive, complex and time-consuming method (58). Several studies have analyzed non-invasive markers of liver steatosis and fibrosis in order to bypass this method. Kulkarni et al. identified a model of several non-invasive parameters that could predict NAFLD induced fibrosis (59). Above all, it seems that a combination of anthropometric, laboratory as well as radiologic methods might improve the practicability and exactitude of diagnosing NAFLD induced fibrosis in pediatric obesity (60, 61).

### Strengths and Limitations

A major strength of this study was the inclusion of both MRI and clamp data in a pediatric cohort. The lack of histological data does not allow us to discriminate between simple steatosis and differing degrees of fibrosis. However, Schwimmer et al. showed a positive correlation between MRI-estimated liver proton density fat fraction and steatosis grades by liver histology (34), which underscores the need to identify patients more readily in clinical practice. In addition, we do not have detailed information on the distribution of ethnicities in our collective, although the majority of our patients is white. This might be important, as differences in IR between ethnicities have been described repeatedly (62, 63). Further, due to its cross-sectional design, our data do not allow us to draw any conclusions on the performance of the SPISE in the evolution of NAFLD longitudinally. However, in order to increase the homogeneity of our cohort, we included data of pubertal patients only (Tanner stages II-IV) and employed robust techniques to assess liver fat content and IR. Due to a limited sample size further studies will be needed in order to validate our findings in larger pediatric cohorts. This would allow more detailed analyses of SPISE cutoffs in children with different pubertal stages and degrees of obesity.

In conclusion, in a clinical setting the early diagnosis of NAFLD is of utmost importance, since its progression to fibrosis has substantial impact on overall morbidity in the pediatric population and morbidity and mortality in later life. Thus, additional simple surrogates of hepatic insulin resistance aiding in the clinical diagnosis of NAFLD are needed. SPISE outperformed established indices of hepatic insulin resistance when compared to M-values derived from hyperinsulinemic clamp tests in both males and females. Although neither index (SPISE, HOMA-IR, HIRI) allowed a differentiation of steatosis-grades within the NAFLD group, SPISE may represent an easy surrogate of hepatic insulin resistance in children with overweight or obesity to be used as a screening tool for hepatic risk assessment on a large scale and in longitudinal studies.

## Data Availability Statement

The original contributions presented in the study are included in the article/supplementary material. Further inquiries can be directed to the corresponding author.

## Ethics Statement

The ethical approvals for the study and necessary amendments were obtained from the ethical committees of Uppsala University (Uppsala Regional Ethics Committee, registration numbers 2010/036 and 2012/318) as well as Salzburg University (Ethics Committee Salzburg 2012/1544). The study was carried out according to the Declaration of Helsinki, following an agreement of good clinical practice. The study physician informed the patients and controls and their families personally and written consent was consequently obtained from children/adolescents and parents separately. Written informed consent to participate in this study was provided by the participants’ legal guardian/next of kin.

## Author Contributions

DF, CHA, HMangge, HManell, PK, SS, DW and KMö conceived and designed the analysis, contributed data, performed the analyses and wrote the paper. KMö, SMB and KMa collected samples and data. PB, AF, JK, HA and AMS collected additional data and contributed to this manuscript.

## Funding

This paper was written as part of the Beta-JUDO (Beta-cell function in Juvenile Diabetes and Obesity) consortium, which was carried out during 2012-2017 within the European FP7-HEALTH-2011-two-stage (project number: 279153). DW has received consultant fees from Novo Nordisk. HA and JK are cofounders, stock owners and employees of Antaros Medical, Mölndal/Sweden.

## Conflict of Interest

The authors declare that the research was conducted in the absence of any commercial or financial relationships that could be construed as a potential conflict of interest.

## Publisher’s Note

All claims expressed in this article are solely those of the authors and do not necessarily represent those of their affiliated organizations, or those of the publisher, the editors and the reviewers. Any product that may be evaluated in this article, or claim that may be made by its manufacturer, is not guaranteed or endorsed by the publisher.
